# The microbiological profile and presence of bloodstream infection influence mortality rates in necrotizing fasciitis

**DOI:** 10.1186/cc10278

**Published:** 2011-06-21

**Authors:** I-Chuan Chen, Wen-Cheng Li, Yu-Cheng Hong, Shian-Sen Shie, Wen-Chih Fann, Cheng-Ting Hsiao

**Affiliations:** 1 Department of Emergency Medicine, Chang Gung Memorial Hospital, No.6, W. Sec., Jiapu Rd., Puzih City, Chiayi County 613, Taiwan; 2 Division of Infectious Diseases, Department of Internal Medicine, Chang Gung Memorial Hospital, No.6, W. Sec., Jiapu Rd., Puzih City, Chiayi County 613, Taiwan; 3 Chang Gung University College of Medicine, No.5, Fusing St., Gueishan Township, Taoyuan County 333, Taiwan; 4 Department of Occupation Medicine, Chang-Gung Memorial Hospital, Keelung Branch, No. 222, Maijin Rd., Keelung, Taiwan

## Abstract

**Introduction:**

Necrotizing fasciitis (NF) is a life threatening infectious disease with a high mortality rate. We carried out a microbiological characterization of the causative pathogens. We investigated the correlation of mortality in NF with bloodstream infection and with the presence of co-morbidities.

**Methods:**

In this retrospective study, we analyzed 323 patients who presented with necrotizing fasciitis at two different institutions. Bloodstream infection (BSI) was defined as a positive blood culture result. The patients were categorized as survivors and non-survivors. Eleven clinically important variables which were statistically significant by univariate analysis were selected for multivariate regression analysis and a stepwise logistic regression model was developed to determine the association between BSI and mortality.

**Results:**

Univariate logistic regression analysis showed that patients with hypotension, heart disease, liver disease, presence of *Vibrio *spp. in wound cultures, presence of fungus in wound cultures, and presence of *Streptococcus *group A, *Aeromonas *spp. or *Vibrio *spp. in blood cultures, had a significantly higher risk of in-hospital mortality. Our multivariate logistic regression analysis showed a higher risk of mortality in patients with pre-existing conditions like hypotension, heart disease, and liver disease. Multivariate logistic regression analysis also showed that presence of *Vibrio *spp in wound cultures, and presence of *Streptococcus *Group A in blood cultures were associated with a high risk of mortality while debridement > = 3 was associated with improved survival.

**Conclusions:**

Mortality in patients with necrotizing fasciitis was significantly associated with the presence of *Vibrio *in wound cultures and *Streptococcus *group A in blood cultures.

## Introduction

Understanding the mechanisms underlying the pathophysiology of an infectious disease and characterization of the causative organism are key to providing better medical or surgical care, preventing complications and initiating early, appropriate antimicrobial treatment. Necrotizing fasciitis (NF), a life-threatening infectious disease with mortality rate ranging from 17% to 34% [1-6] actually refers to a spectrum of diseases where necrosis of deeper soft tissue is driven by an infective microorganism [7]. NF primarily involves the superficial fascia with extensive deterioration of the surrounding tissue. It has been suggested that the rapid, soft tissue necrosis seen in NF is caused by the release of bacterial toxins and enzymes, which may then lead to extensive inflammation, sepsis and multiple organ failure [8]. We previously developed a laboratory risk indicator for necrotizing fasciitis (LRINEC), which is a useful tool to distinguish necrotizing soft tissue infections from other soft tissue infections [9].

The overall annual incidence of necrotizing fasciitis in the USA was reported as 0.04/1,000 people [10]. NF has been classified based on different criteria such as the anatomical level of involvement or the requirement of surgical management [11]. However, it is most convenient to categorize NF based on the microbiological characteristics of the pathogen involved [12-14]. Type 1 necrotizing fasciitis is a polymicrobial infection arising from aerobic and anaerobic bacteria, while Type 2 necrotizing fasciitis is caused by group A *Streptococcus *with or without a coexisting *Staphylococcal *infection. Although necrotizing fasciitis caused by fungi was previously classified under Type 2, fungal necrotizing fasciitis has recently been classified under its own category [15].

Although early diagnosis of NF is key to managing the disease, it is complicated by the fact that NF typically presents with vague, non-specific symptoms. Treatment typically consists of a combination of surgical debridement, antibiotic treatment based on the pathogen and oxygenation of the injured tissue [16]. Immunocompromise, diabetes mellitus, alcoholism, end-stage renal disease, malignancy and chemotherapy have all been suggested as predisposing factors in NF [16]. However, NF can also occur in otherwise healthy adults and is usually precipitated by some form of trauma [17,18].

A number of studies have looked at prognostic factors in NF. Diabetes mellitus was shown to be significantly associated with mortality [16,19]. Our previous study of 128 patients showed that *Vibrio *infection and *Aeromonas *infection were strongly associated with mortality [20]. Mortality rates in NF are also influenced by the presence of bloodstream infection (BSI) [6,12]. However, the impact of individual, causative bacteria in BSI and outcomes has not yet been studied in patients with necrotizing fasciitis.

The primary endpoint of the present study was to characterize the causative microorganism of necrotizing fasciitis. The secondary endpoint was to investigate the correlation between mortality and the causative pathogens of a wound or blood stream infection.

## Materials and methods

This retrospective study enrolled a total of 323 consecutively presenting, necrotizing fasciitis patients who were admitted between January 2002 and September 2005 via the emergency department (ED) of two different hospitals (the Gueishan Chang Gung Medical Center and the Chiayi Chang Gung Memorial Hospital). Out of a total of 354 necrotizing fasciitis patients, 31 patients were excluded from the study due to a lack of records. The study sites included a tertiary academic center, serving a population of approximately one million, with an annual ED census of 180,000 visits per year and a community, university-affiliated hospital, serving a population of approximately 0.3 million, with an annual ED census of 60,000 visits per year. Both hospitals had 24 hour in-house coverage by a senior plastic or orthopedic surgery resident, with an attending plastic or orthopedic surgeon available within 30 minutes.

Inclusion criteria were 1) patients who had undergone surgery and 2) availability of histology and pathology reports. A diagnosis of necrotizing fasciitis is usually difficult and cannot be based solely on microbiological findings. In this study, a diagnosis of necrotizing fasciitis was based on clinical manifestations and surgical findings in our hospital and was confirmed by either clinical, microbiologic, radiographic, gross anatomic findings or histologic examination. Information collected at the time of admission included age, gender, comorbidities, clinical symptoms/signs, site and etiology of infection, emergency department (ED) triage vital signs, laboratory findings at the time of admission and medication being taken at the time of admission. The time from admission to operation, the need for amputation, the duration of hospitalization, and the mortality rates were reviewed and the microbiology of wound culture and blood culture were also documented. The study population was very heterogenous in order to ensure the inclusion of a large number of causative pathogens. Hypotension was defined as a systolic blood pressure < 90 mmHg at ED triage. Positive wound or blood culture was defined as successful culture from the wound or blood within three days. Infection was defined as the presence of a pathogenic microorganism in a wound culture or blood culture or as a clinically suspected infection plus the administration of antibiotics [21,22]. Sepsis was defined by the guidelines of the American College of Chest Physicians/Society of Critical Care Medicine Consensus Conference as an infection plus two systemic inflammatory response syndrome criteria [21,22]. Bloodstream infection (BSI) was defined as a positive growth of bacteria or fungus from at least one blood culture. Following hospital protocol, all patients were administered antibiotics prior to surgery. Once NF was suspected, patients were immediately administered third generation cephalosporin (Ceftriaxone) with metronidazole or clindamycin at the emergency department. Vancomycin was only prescribed at the emergency department if methicillin-resistant *Staphylococcus aureus *(MRSA)-related NF was suspected. The number of debridements was based on the clinical judgment of the surgeon.

All wound cultures obtained aseptically during the initial surgery, were placed in standard aerobic and anaerobic swab and transport tubes and plated within one hour in our laboratory on standard sheep blood agar plates. Chocolate, casein-soy and MacConkey agar media were used for aerobic bacteria and standard anaerobic medium and broth were used for anaerobic bacteria. Plates were incubated at 36°C under aerobic or anaerobic conditions. None of the patients received IVIG or Xigris treatment. The study was approved by the Institute Reviewing Board of Chang Gung Memorial Hospital and informed consents from patients were waived. Laboratory standard protocol for the bacterial culture was followed.

### Statistical analysis

Demographic data were expressed as the mean with standard deviation for numerical data, and n with percentage (%) for contingency data. Microbiologic characteristics were summarized as n (%) for 1) subjects who were positive for wound culture growth, 2) subjects positive for blood culture growth (single etiologic agent) and 3) subjects positive for blood culture growth (multiple etiologic agents). We used the Pearson chi-square test or Fisher's exact test to compare the dispersion in microbiologic characteristics between subjects with single etiologic agent, and those with multiple etiologic agents. We also used Fisher's exact test to compare the association of patients' mortality with microbiologic characteristics. In addition, we performed univariate and multivariate logistic regression analyses to identify the prognostic factors which might be associated with mortality. A *P*-value less than 0.05 was considered statistically significant. All statistical analyses were performed using SPSS 15.0 statistics software (SPSS Inc, Chicago, IL, USA).

## Results

A total of 323 patients with necrotizing fasciitis were enrolled in this study. Table 1 shows the demographic characteristics of the patients. Males comprised 69.3% of the study population (224 males) and the average age was 57.5 years (SD = 15.3). The mortality rate was 16.1% (52/323). We observed that 279 (86.4%) subjects were positive for growth of wound cultures, and 65 (20.1%) subjects were positive for growth of blood cultures (Table 1). Sixty percent of our study population was diabetic and 68.7% of the patients underwent surgery within 24 hours of admission.

**Table 1:** Demographic characteristics of 323 patients with necrotizing fasciitis

Demographic characteristics	(N = 323)
Age^1^, years	57.5 ± 15.3
Gender^2^, male (%)	224 (69.3)
**Pre-existing disease**^2^	
Diabetes mellitus	195 (60.4)
Liver disease	91(28.2)
Renal disease	60 (18.6)
Heart disease	43 (13.3)
Peripheral vascular disease	27 (8.4)
Intravenous drug use	15 (4.6)
**Clinical features**^2^	
Infected site, limbs	276 (85.4)
Tender	261(80.8)
Erythema	252 (78.0)
Presence of sepsis	244 (75.5)
Tachycardia	152 (47.1)
Bullae formation	132 (40.9)
Fever	103 (31.9)
Hypotension	42 (13.0)
**Therapeutic factors and the outcomes**	
Operation within 24 hours^2^	222 (68.7)
Amputation^2^	83 (25.7)
Mortality^2^	52 (16.1)
Number of debridement^1^, number	3.14 ± 2.2
Hospital length of stay^1^, days	35.0 ± 26.1
**Wound culture, positive (%)**	279 (86.4)
**Blood culture, positive (%)**	65 (20.1)

^1,2 ^Data were expressed as mean ± SD for ^1^numerical data, and n (%) for ^2^contingency data.

Table 2 summarizes the microbiologic characteristics of the 279 patients who were positive for growth of wound cultures. Of the 279 patients, there were 126 patients with a single etiologic agent, and 153 patients with multiple etiologic agents. In the single etiology group, we found a significantly higher dispersion of subjects with Methicillin-susceptible *S*. *aureus *(MSSA) when compared with the multiple etiology group. *Vibrio *species, methicillin-resistant *Staphylococcus aureus *(MRSA), group A beta-hemolytic *Streptococcus *and *Klebsiella pneumoniae *were other the most common pathogens in the single etiology group.

**Table 2:** Microbiologic characteristics of 279 patients who had positive growth of wound cultures

Variable	Single etiologic agent (*n *= 126)	Multiple etiologic agents (*n *= 153)	*P*-value
***Gram-positive cocci***			
**Aerobic**			
*Staphylococcus aureus*			
Methicillin-susceptible *S. aureus*	27 (21.4)	15 (9.8)	0.007*
Methicillin-resistant *S. aureus*	20 (15.9)	34 (22.2)	0.182
Coagulase-negative *Staphylococcus*	2 (1.6)	11 (7.2)	0.042*
*Streptococcus*			
Beta-hemolytic (Group A)	13 (10.3)	24 (15.7)	0.188
Beta-hemolytic (Group B)	2 (1.6)	4 (2.6)	0.693
Non-group A or B	-	2 (0.7)	-
*S. viridans*	2(1.6)	36 (23.5)	<0.001*
*Enterococcus faecalis*	1(0.8)	45 (29.4)	<0.001*
**Anaerobic**			
*Peptostreptococcus*	-	38 (13.6)	-
***Gram-positive rods***			
**Aerobic**			
*Corynebacterium*	-	8 (2.9)	-
**Anaerobic**			
*Clostridium spp*.	-	5 (1.8)	-
***Gram negative rods***			
**Aerobic**			
*Acinetobacter baumannii*	3 (2.4)	20 (13.1)	0.001*
*Aeromonas *spp.	11 (8.7)	11 (7.2)	0.635
*Citrobacter *spp.	2 (1.6)	9 (5.9)	0.119
*E.coli*.	2 (1.6)	35 (22.9)	<0.001*
*Enterobacter *spp.	1 (0.8)	19 (12.4)	<0.001*
*Klebsiella pneumoniae*	13 (10.3)	28 (18.3)	0.061
*Pseudomonas aeruginosa*	3 (2.4)	16 (10.5)	0.008*
*Vibrio *spp.	21 (17.5)	-	-
*Eikenella corrodens*	-	4 (2.6)	-
*Klebsiella oxytoca*	-	3 (2.0)	-
*Proteus mirabillis*	-	23 (15.0)	-
*Serratia marcescens*	-	7 (4.6)	-
Other gram negative rods	1 (0.8)	15 (9.2)	0.002*
**Anaerobic**			
*Prevotella *spp.	2 (1.6)	10 (6.5)	0.071
*Bacteroides fragilis*	-	15 (9.8)	-
*Fusobacterium*	-	1 (0.7)	-
*Veillonella*	-	3 (2.0)	-
***Fungus***	-	6 (3.9)	-

Data were summarized as n (%) for the subjects with positive growth of wound culture by single etiologic agent, and multiple etiologic agent, respectively.

* *P *<0.05 significantly different between single, and multiple etiologic agents, *P*-values were derived through Pearson Chi-square test, or Fisher's exact test.

The multiple etiology group showed a significantly higher number of patients with Gram positive cocci such as *Enterococcus fecalis *and *S. viridians *and Gram negative rods such as *E. coli*., *Enterobacter *spp., *Acinetobacter baumannii*, and *Pseudomonas aeroginosa *when compared with the single etiology group.

The microbiologic characteristics of 65 patients who were positive for growth of blood cultures are summarized in Table 3. Of the 65 patients, there were 61 patients with a single etiologic agent, and 4 patients with multiple etiological agents. In the single etiology group, we found a significantly lower number of patients with *E. coli*. when compared with the multiple etiology group, while *Vibrio *species, MSSA and MRSA were the most common pathogens in this group.

**Table 3:** Microbiologic characteristics of 65 patients with positive growth of blood cultures

Variable	Single etiologic agent (*n *= 61)	Multiple etiologic agents (*n *= 4)	*P*-value
***Gram-positive bacteria***			
**Aerobic**			
*Staphylococcus aureus*			
Methicillin-susceptible *S. aureus*	9 (14.8)	1 (25.0)	0.496
Methicillin-resistant *S. aureus*	7 (11.5)	-	-
*Streptococcus*			
Beta-hemolytic (Group A)	5 (8.2)	-	-
Beta-hemolytic (Group B)	1 (1.6)	1 (25.0)	0.120
**Anaerobic**			
*Peptostreptococcus*	1 (1.6)	1 (25.0)	0.120
**Gram negative bacteria**			
**Aerobic**			
*Acinetobacter baumannii*	2 (3.3)	-	-
*Aeromonas *spp.	6 (9.8)	-	-
*Citrobacter *spp.	1 (1.6)	-	-
*E.coli*	3 (4.9)	2 (50.0)	0.027*
*Enterobacter *spp.	1 (1.6)	1 (25.0)	0.120
*Klebsiella pneumoniae*	2 (3.3)	1 (25.0)	0.176
*Proteus mirabillis*	1 (1.6)	-	-
*Vibrio *spp.	20 (32.8)	-	-
Other gram negative rods	1 (1.6)	-	-
**Anaerobic**			
*Prevotella *spp.	1 (1.6)	-	-
*Bacteroides fragilis*	-	1 (25.0)	-

Data were summarized as n (%) for subjects with positive growth of blood culture (single etiologic agent or multiple etiologic agent).

* *P *<0.05 denotes a significant difference between single, and multiple etiologic agents, *P*-values were derived through Fisher's exact test.

We investigated the association of mortality with microbiological profile in the 279 patients who were positive for growth of wound cultures (Table 4) and in the 65 patients who were positive for growth of blood cultures (Table 5). Of the 38 patients with Gram negative bacterial BSI in our study, 81.6% had sepsis and 50% had hypotension at ER triage (data not shown). We showed that mortality was significantly associated with the presence of *Clostridium *spp. (*P *= 0.035) and *Vibrio *spp. (*P *<0.001) in wound cultures.

**Table 4:** Association of mortality with microbiologic characteristics of 279 patients with positive growth of wound cultures

Variable	Survival (*n *= 232)	Death (*n *= 47)	*P*-value
***Gram-positive cocci***			
**Aerobic**			
*Staphylococcus aureus*			
Methicillin-susceptible *S. aureus*	39 (16.8)	3 (6.4)	0.075
Methicillin-resistant *S. aureus*	47 (20.3)	7 (14.9)	0.543
Coagulase-negative *Staphylococcus*	12 (5.2)	1 (2.1)	0.703
*Streptococcus*			
Beta-hemolytic (Group A)	32 (13.8)	5 (10.6)	0.645
Beta-hemolytic (Group B)	4 (1.7)	2 (4.3)	0.267
Non-group A or B			
*S. viridans*	33 (14.2)	5 (10.6)	0.644
*Enterococcus faecalis*	37 (15.9)	9 (19.1)	0.666
**Anaerobic**			
*Peptostreptococcus*	30 (12.9)	8 (17.0)	0.485
***Gram-positive rods***			
**Aerobic**			
*Corynebacterium*	7 (3.0)	1 (2.1)	1.000
**Anaerobic**			
*Clostridium *spp.	2 (0.9)	3 (6.4)	0.035*
***Gram negative rods***			
**Aerobic**			
*Acinetobacter baumannii*	20 (8.6)	3 (6.4)	0.776
*Aeromonas *spp.	18 (7.8)	4 (8.5)	0.773
*Citrobacter *spp.	10 (4.3)	1 (2.1)	0.697
*E.coli*.	29 (12.5)	8 (17.0)	0.478
*Enterobacter *spp.	15 (6.5)	5 (10.6)	0.349
*Klebsiella pneumoniae*	36 (15.5)	5 (10.6)	0.501
*Pseudomonas aeruginosa*	16 (6.9)	3 (6.4)	1.000
*Vibrio *spp.	11 (4.7)	11 (23.4)	<.001*
*Eikenella corrodens*	4 (1.7)	-	-
*Klebsiella oxytoca*	3 (1.3)	-	-
*Proteus mirabillis*	23 (9.9)	-	-
*Serratia marcescens*	7 (3.0)	-	-
Other gram negative rods	12 (5.2)	3 (6.4)	0.724
**Anaerobic**			
*Prevotella *spp.	12 (5.2)	-	-
*Bacteroides fragilis*	11 (4.7)	4 (8.5)	0.292
*Fusobacterium*	1 (0.4)	-	-
*Veillonella*	2 (0.9)	1 (2.1)	0.426
***Fungus***	**5 (2.2)**	1 (2.1)	**1.000**

Data were summarized as n (%) for subjects with positive growth of wound culture. Subjects were classified into "Death" and Survival" categories. * *P *<0.05 denotes a significant difference between the Death, and Survival groups. *P*-values were derived through Fisher's exact test.

**Table 5:** Microbiologic characteristics of 65 patients with positive growth of blood culture by patients' mortality

Variable	Survival (*n *= 41)	Death (*n *= 24)	*P*-value
***Gram-positive bacteria***			
**Aerobic**			
*Staphylococcus aureus*			
Methicillin-susceptible *S. aureus*	10 (24.4)	-	-
Methicillin-resistant *S. aureus*	5 (12.2)	2 (8.3)	1.000
*Streptococcus*			
Beta-hemolytic (Group A)	2 (4.9)	3 (12.5)	0.350
Beta-hemolytic (Group B)	1 (2.4)	1 (4.2)	1.000
**Anaerobic**			
*Peptostreptococcus*	2 (4.9)	-	-
**Gram negative bacteria**			
**Aerobic**			
*Acinetobacter baumannii*	-	2 (8.3)	-
*Aeromonas *spp.	3 (7.3)	3 (12.5)	0.662
*Citrobacter *spp.	1 (2.4)		
*E.coli*	3 (7.3)	2 (8.3)	1.000
*Enterobacter *spp.	1 (2.4)	1 (4.2)	1.000
*Klebsiella pneumoniae*	3 (7.3)		
*Proteus mirabillis*		1 (4.2)	
*Vibrio *spp.	11 (26.8)	9 (37.5)	0.412
Other gram negative rods	1 (2.4)	-	-
**Anaerobic**			
*Prevotella *spp.	1 (2.4)	-	-
*Bacteroides fragilis*	-	1 (4.2)	-

Data were summarized as n (%) for the subjects with positive growth of blood culture by patients' mortality, respectively.

*P*-values were derived through Fisher's exact test.

We used univariate and multivariate logistic regression analyses to look at the relationship between mortality and the presence of specific microorganisms in wound and blood cultures. We also investigated the relationship of mortality with other prognostic factors (Tables 6 and 7). We showed a higher risk of mortality in patients with hypotension (OR: (95% confidence interval (CI)) = 7.07 (3.48 to 14.37), *P*<0.001), heart disease (OR: (95% CI) = 3.52 (1.72 to 7.20), *P *= 0.001), liver disease (OR. (95% CI) = 2.37 (1.28 to 4.37), *P *= 0.006), presence of *Vibrio *spp. in wound cultures (OR: (95% CI) = 6.37 (2.58 to 15.57), *P *<0.001), presence of fungus (candida) in wound cultures (OR: (95% CI) = 6.34 (2.58 to 15.57, *P *<0.001); presence of *Streptococcus *group A in blood cultures (OR: (95% CI) = 8.24 (1.34 to 50.56), *P *= 0.023), presence of *Aeromonas *spp in blood cultures (OR: (95% CI) = 5.47 (1.07 to 27.89), *P *= 0.041), and presence of *Vibrio *spp. in blood cultures (OR: (95% CI) = 4.95 (1.94 to 12.64), *P *= 0.001] (Table 6).

**Table 6:** Univariate logistic regression analysis of mortality related to multiple prognostic factors; (N = 323)

Variables	Survival (*n *= 271)	Death (*n *= 52)	OR (95% CI.)^3^	*P*-value^4^
**Age^1^, years**	57.4 ± 15.5	58.4 ± 14.2	1.00 (0.98 to 1.02)	0.607
**Gender^2^, males (%)**	192 (70.8)	32 (61.5)	1.24 (0.64 to 2.41)	0.525
**Pre-existing disease^2^**				
**Fever, n (%)**	85 (31.4)	18 (34.6)	1.16 (0.62 to 2.17)	0.645
**Tachycardia**	126 (46.5)	26 (50.0)	1.05 (0.58 to 1.90)	0.872
**Hypotension**	30 (11.1)	12 (23.1)	7.07 (3.48 to 14.37)	<.001*
**Diabetes mellitus**	167 (61.6)	28 (53.8)	1.06 (0.58 to 1.95)	0.851
**Heart disease**	37 (13.7)	6 (11.5)	3.52 (1.72 to 7.20)	0.001*
**Peripheral vascular disease**	23 (8.5)	4 (7.7)	0.39 (0.09 to 1.71)	0.214
**Renal disease**	49 (18.1)	11 (21.2)	1.59 (0.79 to 3.21)	0.196
**Liver disease**	74 (27.3)	17 (32.7)	2.37 (1.28 to 4.37)	0.006*
**Intravenous drug use**	14 (5.2)	1 (1.9)	0.36 (0.05 to 2.80)	0.329
**Clinical features^2^**				
**Affected site (limbs)**	228 (84.1)	48 (92.3)	1.11 (0.47 to 2.64)	0.808
**Bullae formation**	109 (40.2)	23 (44.2)	1.18 (0.65 to 2.15)	0.590
**Operation within 24 hours**	187 (69.0)	35 (67.3)	0.68 (0.35 to 1.26)	0.224
**Amputation**	70 (25.8)	13 (25.0)	1.86 (0.99 to 3.50)	0.053
**Debridement > = 3 times**	157 (57.9)	11 (21.2)	0.19 (0.10 to 0.39)	<.001*
**Multiple Etiologic agent in Wound culture^2^, n (%)**	129 (47.6)	24 (46.2)	0.94 (0.52 to 1.71)	0.848
**Gram positive organism in wound culture^2^**				
***Streptococcus *group A**	32 (11.8)	5 (9.6)	0.79 (0.29 to 2.14)	0.650
***Streptococcus *group B**	4 (1.5)	2 (3.8)	2.67 (0.48 to 14.97)	0.264
**Methicillin-susceptible** ***S. aureus***	39 (14.4)	3 (5.8)	0.36 (0.11 to 1.23)	0.103
**Methicillin-resistant *S. aureus***	47 (17.3)	7 (13.5)	0.74 (0.31 to 1.74)	0.493
**Gram negative organism in wound culture^2^**				
***Acinetobacter baumannii***	20 (7.4)	3 (5.8)	0.77 (0.22 to 2.69)	0.680
***Aeromonas *spp**.	18 (6.6)	4 (7.7)	1.17 (0.38 to 3.61)	0.783
***E.coli***	29 (10.7)	8 (15.4)	1.52 (0.65 to 3.54)	0.334
***K.pneumoniae***	36 (13.3)	5 (9.6)	0.69 (0.26 to 1.86)	0.469
***Proteus mirrabilis***	23 (8.5)	0 (0)	-	-
***Pseudomonas *spp**.	16 (5.9)	3 (5.8)	0.98 (0.27 to 3.48)	0.970
***Vibrio *spp**.	11 (4.1)	11 (21.2)	6.37 (2.58 to 15.57)	<.001*
**Fungus in wound culture^2^**	6 (2.2)	1 (1.9)	6.34 (2.58 to 15.57)	<.001*
**Multiple Etiologic agent in blood culture^2^, n (%)**	3 (1.1)	1 (1.9)	1.75 (0.18 to 17.17)	0.630
**Gram positive organism in blood culture^2^**				
***Streptococcus *group A**	2 (0.7)	3 (5.8)	8.24 (1.34 to 50.56)	0.023*
***Streptococcus *group B**	1 (0.4)	1 (1.9)	5.29 (0.33 to 86.01)	0.241
**Methicillin-susceptible** ***S. aureus***	10 (3.7)	0 (0)	-	-
**Methicillin-resistant *S. aureus***	5 (1.8)	2 (3.8)	2.13 (0.40 to 11.27)	0.375
**Gram negative organism in blood culture^2^**				
***Acinetobacter baumannii***	0 (0)	2 (3.8)	-	-
***Aeromonas *spp**.	3 (1.1)	3 (5.8)	5.47 (1.07 to 27.89)	0.041*
***E.coli***	3 (1.1)	2 (3.8)	3.57 (0.58 to 21.93)	0.169
***Proteus mirrabilis***	0 (0)	1 (1.9)	-	-
***Vibrio *spp**.	11 (4.1)	9 (17.3)	4.95 (1.94 to 12.64)	0.001*

^1,2 ^Data were summarized as mean ± SD, and n (%) for continuous, and categorical variables for subjects by survival status, respectively.

^3 ^OR: (95% CI), Odds ratio with 95% confidence interval were derived through univariate logistic regression model.

^4 ^*P*-values from univariate logistic regression analysis, were used to identify the significance of respective odds ratio (OR) *P <0.05.

**Table 7:** Multivariate logistic regression analysis of mortality related to multiple prognostic factors; (N = 323)

Variables	OR (95% CI)^1^	*P*-value^2^
**Age, years**	0.99 (0.97 to 1.02)	0.607
**Gender, males (%)**	1.16 (0.50 to 2.67)	0.731
**Pre-existing disease**		
**Hypotension**	5.68 (2.21 to 14.59)	<0.001*
**Heart disease**	2.89 (1.09 to 7.62)	0.033*
**Liver disease**	2.54 (1.10 to 5.87)	0.029*
**Clinical features**		
**Operation within 24 hours**	0.59 (0.27 to 1.29)	0.182
**Debridement > = 3 times**	0.19 (0.08 to 0.43)	<.001*
**Gram negative organism in wound culture**		
***Vibrio *spp**.	5.60 (1.82 to 17.25)	0.003*
**Fungus in wound culture**	0.52 (0.04 to 7.54)	0.632
**Gram positive organism in blood culture**		
***Streptococcus *group A**	15.93 (1.95 to 130.34)	0.010*
**Gram negative organism in blood culture**		
***Aeromonas *spp**.	3.50 (0.46 to 26.76)	0.227
***Vibrio *spp**.	1.19 (0.29 to 4.92)	0.806

^1 ^Odds ratio with 95% confidence interval (OR: (95% CI) were derived through multivariate logistic regression model.

^2 ^*P*-values from multivariate logistic regression analysis, were used to identify the significance of respective OR.*P <0.05.

Our data also showed a significantly lower risk of mortality in subjects with debridement > = 3 times (OR: (95% CI) = 0.19 (0.10 to 0.39), *P *<0.001] (Table 6).

We used multivariate logistic regression analysis to investigate the prognostic factors which were significant in the univariate logistical analysis. Since a 24-hour or more delay until the operation is a risk factor for death, the multivariate analysis was performed controlling for subjects with a 24-hour or more delay until the operation. Our data showed a higher risk of mortality in patients with pre-existing diseases, (hypotension, heart disease, and liver disease). The presence of *Vibrio *spp in wound cultures, and the presence of *Streptococcus *group A in blood cultures also increased the risk of mortality whereas patients with debridement > = 3 times had a lower risk of mortality (Table 7).

We performed univariate analysis to investigate the association between positive blood cultures, positive wound cultures and survival (Table 8). We showed that patients with positive blood stream infections had a 2.26 higher incidence of death. However, we found no significant association between positive wound cultures and mortality.

**Table 8:** Univariate analysis of mortality related to blood stream infection and positive wound cultures

Variables	Survival (*n *= 271)	Death (*n *= 52)	OR (95% CI.)^3^,	*P*-value^4^
**Positive blood stream infection**	48 (17.7)	17 (32.7)	2.26 (1.17 to 4.36)	0.015
**Wound culture positive**	235 (86.7)	44 (84.6)	0.84 (0.37 to 1.93)	0.686

## Discussion

In this study, we showed that patients with necrotizing fasciitis who were positive for *Vibrio *spp in wound culture, or *Streptococcus *group A in blood culture were at higher risk for mortality. The presence of certain pre-existing conditions, such as diabetes mellitus, hypotension, heart disease or liver disease, were also predictors of higher mortality, while debridement > = 3 times was associated with a lower risk of mortality.

NF is caused by a number of different pathogens. Our study population had a predominance of polymicrobial infections (54.8%, 153/279). However, our data agreed with previous studies that showed that infections caused by single organisms were not uncommon [20,23]. We found that MSSA, MRSA and *Vibrio *spp. were the most common pathogens in the single etiology group. *Staphylococcus *aureus was recently shown to be present in 18.9% of patients with necrotizing fasciitis of limbs [24] and was the most frequently (21.7%) isolated bacteria in NF patients [11]. Wound cultures of our study patients showed 1) a predominance of Methicillin-susceptible *Staphylococcus aureus*, 2) a significantly lower occurrence of Gram positive cocci such as coagulase-negative *Staphylococcus*, *S*. *viridans *and *Enterococcus fecalis *and 3) a significantly lower occurrence of Gram negative rods, such as *Acinetobacter baumannii*, *Enterobacter *spp., *E. coli *and *Pseudomonas aeroginosa *in the single etiology groups. Blood cultures showed a significantly lower occurrence of *E. coli *in the single etiology group. It would be interesting to dissect the mechanisms underlying these differences in microbiological profiles between the single etiology groups and the multiple etiology groups.

Interestingly, we showed that *Vibrio *spp., previously considered a rare cause of necrotizing fasciitis, accounted for 21 cases within our study population. *Vibrio *spp. are known to flourish in warm saltwater at temperatures between 20°C and 68°C and are found mainly in Australia, Asia (Thailand, Taiwan and Hong Kong), the Gulf of Mexico and South America as described by the Centers for Disease Control [25]. These findings are consistent with the fact that most of our study patients with *Vibrio *infections were from the Chiayi Chang Gung Memorial Hospital study site located close to two local harbors. *Vibrio *spp. (usually *V. vulnificus*) is known to cause severe necrotizing wound infection, necrotizing fasciitis and sepsis [23,26], resulting in a separate classification of Type 3 necrotizing fasciitis caused by marine *Vibrio *spp. [13,27]. Ingestion of *V. vulnificus *in the form of contaminated seafood, such as raw fish, raw oysters or from open wounds that have been exposed to seawater, can lead to bloodstream infection and NF, especially in immunocompromised patients, with a mortality rate of about 50% [23]. Our results were consistent with these data and showed that all our study patients with *Vibrio *infection (21 cases) either had a history of an open wound which was exposed to seawater or a history of eating raw seafood. Twenty of these patients developed bloodstream infections.

Clostridial infections, although rare, are fulminant and fatal infections resulting in myonecrosis and are an independent predictor for limb loss and mortality [11]. Our results were consistent with these data and showed higher mortality rates in patients infected with *Clostridium *spp. However, the number of Clostridial infections in our study was smaller (five cases) than previously published reports. Clostridial infections were previously shown to be significantly associated with intravenous drug use. The small number of NF patients with a history of intravenous drug use in our study (15 cases), could explain the smaller number of clostridial infections in our study population.

Bloodstream infection is associated with increased morbidity, duration of hospital stay and mortality [28,29] and was previously shown to be a significant risk factor for mortality in NF patients [6,12,30,31]. Our data showed that patients with positive blood stream infections had a 2.26-fold higher incidence of death, while there was no significant association between positive wound cultures and mortality. Despite recent advances in medical care, invasive Group A Streptococcus-induced NF patients have high mortality rates (ranging from 18% to 34%) [32,33]. The reported rates of bacteremia with Group A streptococci range from 46 to 85% [34-36]. Our results showed that patients with Group A *Streptococcus *in blood cultures had significantly higher mortality rates when compared with patients negative for Group A *Streptococcus*. This is possibly due to the presence of toxic shock/myositis, reflecting the amount of circulating exotoxin. Our multivariate analysis showed that increased mortality was associated with the presence of *Vibrio *spp. in wound cultures and *Streptococcus *Group A in blood cultures.

Independent predictors of mortality in NF patients, such as age, gender, existence of comorbidities or serum lactate and sodium levels at the time of admission, have not proved definitive [11,37]. However, early surgical intervention is associated with decreased mortality rates [2,38,39]. A study of mortality in NF patients showed a significantly longer average time (90 hours) from admission to surgery among non-survivors when compared with survivors (25 hours) [2]. We previously showed that a prolonged ED boarding stay was associated with increased mortality, while early operation (within 24 hours of admission) was associated with decreased mortality [40]. Our present data on patient mortality were consistent with some previous studies [1,11,37] but were significantly lower than other previously reported data [2-6]. Most of the NF patients in our study (68.7%) received surgical intervention within 24 hours of admission. This may explain 1) the lack of a significant association between the time of surgical intervention and outcomes and 2) the lower mortality seen in our patients.

The retrospective nature of our study is one of the major limitations. We are aware that reviewed medical charts may not contain all variables of interest and may have inconsistent descriptions. In order to overcome these issues, we had all the charts reviewed by two principal investigators. Furthermore, we used standardized chart review procedures to ensure the quality of data collection and to increase the validity and reliability of the data collected. Despite these limitations, ours is the first multi-institutional study, assessing the microbiologic aspects of necrotizing fasciitis in a large number of NF patients and identifying the association between the microbiological characteristics of wound and blood cultures and mortality. These data are key in designing earlier treatment options and more efficient prognostic strategies for the management of NF.

## Conclusions

We characterized the causative microorganisms in a cohort of 323 necrotizing fasciitis patients and determined the relationship between mortality and the causative pathogens of a wound or blood stream infection in these patients.

We showed that hypotension, heart disease, liver disease, the presence of *Vibrio *spp. in wound cultures, presence of fungus in wound cultures and presence of *Streptococcus *group A, *Aeromonas *spp. or *Vibrio *spp. in blood cultures were associated with a higher risk of mortality. Debridement > = 3 times and early surgical intervention were associated with a lower risk of mortality.

## Key messages

• NF is caused by a number of different pathogens.

• Hypotension, heart disease and liver disease were associated with higher mortality in NF patients.

• Presence of *Vibrio *spp in wound cultures, and *Streptococcus *Group A in blood cultures were associated with a high risk of mortality.

• Patients with positive blood stream infections had a 2.26-fold higher incidence of death, while there was no significant association between positive wound cultures and mortality.

• Debridement > = 3 times had a lower risk of mortality.

## Abbreviations

BSI: bloodstream infection; ED: emergency department; LRINEC: laboratory risk indicator for necrotizing fasciitis; MRSA: methicillin-resistant *Staphylococcus aureus; *MSSA: methicillin-susceptible *S*. *aureus*; NF: necrotizing fasciitis.

## Competing interests

The authors declare that they have no competing interests.

## Authors' contributions

WCL and YCH conceived the study, designed the method, supervised the conduct of the data collection, undertook recruitment of participating centers and patients and managed the data, including quality control. ICC conceived the study, designed the method, supervised the conduct of the data collection, undertook recruitment of participating centers and patients and managed the data, including quality control, provided statistical advice on study design and analyzed the data and drafted the manuscript. SSS conceived the study, designed the method, supervised the conduct of the data collection, provided statistical advice on study design and analyzed the data. CTH supervised the conduct of the data collection, chaired the data oversight committee and drafted the manuscript. WCF undertook recruitment of participating centers and patients and managed the data, including quality control and all authors contributed substantially to its revision. ICC and CTH take responsibility for the paper as a whole. All the authors have read and approved the final version of the manuscript.
